# Mortality trends and causes of death among HIV positive patients at Newlands Clinic in Harare, Zimbabwe

**DOI:** 10.1371/journal.pone.0237904

**Published:** 2020-08-27

**Authors:** Cleophas Chimbetete, Tinei Shamu, Maroussia Roelens, Sandra Bote, Tinashe Mudzviti, Olivia Keiser

**Affiliations:** 1 Newlands Clinic, Harare, Zimbabwe; 2 Institute of Global Health, University of Geneva, Geneva, Switzerland; 3 School of Pharmacy, University of Zimbabwe, Harare, Zimbabwe; University of Pittsburgh, UNITED STATES

## Abstract

**Background:**

We report trends in mortality patterns and causes among HIV positive patients, who initiated antiretroviral therapy (ART), at an urban clinic in Harare, Zimbabwe.

**Methods:**

A retrospective cohort study was conducted in which routinely collected data for patients enrolled and followed up between February 2004 and December 2017 were assessed. Patients follow up was from the day of the treatment initiation until exit by death, transfer out or loss to follow up. Two doctors categorized causes of death (COD) as tuberculosis (TB), communicable AIDS, non-communicable diseases (NCDs), malignancies, others and unknown. We used competing risk survival analysis, first to estimate all-causes and cause-specific mortality rates over time, and then to assess risk factors of different causes of death.

**Results:**

A total of 4 868 patients were followed up for 27 527 person years (PY). Among the 506 patients who died, COD was unknown for 76 patients (15%) and common COD were TB (n = 71, 14%), Malignancies (n = 54, 10.7%) Meningitis (n = 39, 7.7%) and NCDs (n = 60, 11.9%). 49.4% of the deaths were within the first year of starting ART. Median age at death was 36 years (IQR:19–46). There was a near threefold increase in proportion of deaths due to NCDs and malignancies over the period of follow up. Low baseline CD4 cell count and WHO stages 3 & 4 were significant risk factors for all-cause mortality.

**Conclusions:**

TB remains the leading cause of death among HIV infected people. Deaths due to NCDs and malignancies increased over time. ART facilities need to incorporate management of NCDs including cancer as part of comprehensive care of PLHIV to reduce mortality.

## Introduction

Increased availability of antiretroviral therapy (ART) has led to a sustained decline in mortality among people living with human immunodeficiency virus (HIV) infection (PLHIV) or acquired immunodeficiency syndrome (AIDS). Consequently this has led to an improvement in the life expectancy of PLHIV approaching that of the HIV uninfected population in the developed world. [1,2]. Although data from developing world are sparse, available data has shown that mortality rates among HIV positive people has declined substantially as ART programs expand [3–5]. HIV infection has become a chronic manageable disease rather than an untreatable fatal illness as before. Causes of death (COD) among HIV-infected patients need to be studied and assessed in order to define appropriate intervention strategies, to improve medical care of patients and further reduce mortality from both HIV and none-HIV associated causes.

In Zimbabwe, the number of patients receiving ART between 2004 and 2016 increased from a few patients to approximately a million [UNAIDS Country Report 2016]. National ART coverage increased from 7% in 2005 [6] to 84.2% (adults) and 89.5% (children) in 2017 [Zimbabwe National & Sub-national HIV Estimates 2017]. Improvements in diagnostic tests for common opportunistic infections such as tuberculosis (TB) using nucleic acid amplification tests (NAAT) and urine lipoarabinomannan testing continue to be developed. Recently, screening for Cryptococcal infection among severely immunosuppressed (CD4<100 cells/ mm^3^) ART naïve patients have been adopted by the World Health Organisation (WHO) [7]. These measures plus the improved availability of ART medicines with better side effect profiles have contributed to improved survival of HIV infected individuals in sub-Saharan Africa (SSA) [8].

Previous reports show that the majority of deaths among HIV infected patients in SSA have been due to HIV associated infections and malignancies [9,10]. As patients survive longer on ART in this changing environment, it is anticipated that there will be a change in the contribution of different illnesses to mortality. ART associated side effects and HIV associated chronic inflammation are additional factors that may influence causes of death among HIV infected individuals. In developed countries where ART has been available for a longer time, there has been a shift towards causes of death that are not directly related to the immunosuppression caused by HIV [11,12]. Awareness of such a changing pattern in the SSA setting is of importance for patient care and resource allocation. Monitoring causes of death in HIV-positive people enables appropriate targeting of interventions to improve the quality of patient care and reduce avoidable mortality.

We retrospectively reviewed the outcomes, calculated the mortality rates, and analyzed the COD in HIV-infected inpatients at a medical centre for HIV infection and AIDS in Harare, Zimbabwe from 2004 to 2017. Furthermore, we assessed the risk factors for mortality among PLHIV. Previously we have looked at ten-year outcomes of patients that initiated ART at NC between 2004 and 2006 [13]. This report gives further details on mortality causes, trends and associated risk factors for death among all patients who initiated ART at NC between 2004 and 2017. Furthermore, this report is justified by the need for health workers to remain vigilant to the changing mortality trends among HIV infected patients.

## Methods

### Study setting

NC is an HIV management clinic in Harare Zimbabwe with approximately 6000 patients in care as of 31 December 2017. The clinic was founded in 2004 and is operated by the Ruedi Luethy Foundation. Details of the clinic operations are described elsewhere [13]. The Clinic offers comprehensive HIV care using Zimbabwe national treatment guidelines [14]. The clinic is a national reference centre for the management of complicated HIV cases e.g. patients failing second-line ART. It therefore admits both ART naïve (HIV positive patients who have never taken ART) and experienced patients (HIV positive patients who have been previously exposed to ART for treatment). The clinic adheres to national guidelines for managing HIV positive patients. These guidelines have been revised in line with guidance from WHO. Major changes in the guidelines have been the criteria to commence ART which have changed from having a CD4 cell count below 200 cells/mm^3^ or WHO stage 3 and 4 in 2004 to 2010. Between 2010 and 2013, ART initiation criteria were a CD4 count below 350 cells/mm3 or WHO stages 3 and 4. From 2013 to 2015 criteria changed to a CD4 cell count below 500 cells/mm^3^ or WHO stage 3 and 4. In 2015 it was recommended that all patients start lifelong ART irrespective of CD4 cell count or clinical stage The clinic began offering routine HIV viral load testing for patient monitoring in 2013.

### Study procedure

We conducted a retrospective cohort study in which routinely collected data for ART naive patients enrolled and followed up between 01 February 2004 and 31 December 2017 were abstracted from the clinic’s electronic database. Variables abstracted were gender, dates of birth, initial visit, ART commencement, death and exit from the cohort, baseline WHO stage, CD4 cell counts, HIV viral loads (VL) and outcome (patient still in care, transferred out, lost to follow up, or deceased). Loss to follow-up was defined as not attending a scheduled clinic visit and not being contactable by the clinic ninety days thereafter. The CD4 cell count and HIV viral load results closest to death were labelled as last CD4 count and viral load respectively. Initiating HIV care was defined as enrolment into the NC program and ART initiation was defined as the date ART was commenced. Being ART naïve referred to patients who had never taken ART for HIV treatment and ART experienced referred to those who had taken ART for treatment of their HIV infection.

We included all ART naive patients with a diagnosis of HIV infection who attended an “initial visit” and were commenced on ART at NC.

An “initial visit” at Newlands Clinic is the first clinic visit during which the clinicians perform a complete physical examination, assign the baseline WHO stage, begin preparation of the patient for ART and conduct baseline laboratory investigations for all patients enrolled into care. COD was either abstracted from the electronic clinic records when it was available or assigned by consensus of the reviewing two doctors (SB and CC). The COD in the clinic records was assigned by the last attending doctor at the place of death and was documented on the death certificate. The two study doctors only assigned COD to patients who did not have documented COD after reviewing all available clinic notes. Causes of death were then grouped as communicable AIDS related illness (ARI), malignancies, chronic non-communicable diseases (NCD), non-AIDS related illness, unknown or other, as shown in Table 1. Advanced HIV disease was assigned as a cause of death to patients who had no documented COD, had no clear clinical diagnosis documented in their clinic notes, for which the study doctors could not establish a specific cause of death after reviewing clinic notes, had very low CD4 cell counts (<200 cells/mm^3^) and died within 3 months of initiating ART..

**Table 1:** Assignment of causes of death into categories.

Group	Causes of death
Communicable AIDS Related Illness	- Pulmonary tuberculosis
	- Advanced HIV
	- Extrapulmonary tuberculosis
	- Meningitis
	- Mycobacterium Avium Complex infection
	- Respiratory infection
	- Diarrhoea
Malignancies	- Kaposi’s Sarcoma
	- Cervical Cancer
	- Non-Hodgkins Lymphoma
	- Other Cancer
Chronic Non-communicable Diseases	- Hypertension
	- Diabetes
	- Renal disease
	- Cardiovascular
	- Cerebrovascular
	- Chronic respiratory disease
	- Gastrointestinal and Hepatic disease
	- Neurological/Psychiatric
Non-AIDS Related Illness	- Malaria
Other	- Pregnancy related
	- Accident
	- Suicide
	- Post-operative
	- Pulmonary embolism
	- Drug toxicity
	- Anaemia
	- Other

### Ethical approval

This study was approved by the Medical Research Council of Zimbabwe, approval number: MRCZ/E/195.

### Data analysis

Patients follow up, measured in years, was commenced from the day of the ART commencement until exit by death, transfer out or loss to follow up. Lost to follow-up were those who did not attend their last scheduled visit and could not be contacted for over 120 days thereafter. Deceased patients were those whose death had been communicated to the clinic either by evidence of a death certificate or information from their next of kin. Transferred out were those that were provided with an official transfer letter to a different ART services facility.

For patients still in care at the time of data abstraction, follow up time was censored at 31 December 2017. First, we analysed all-cause mortality. We calculated cumulative incidence of mortality, and investigated predictors of all-cause mortality using Fine and Gray proportional Hazards Regression [15], considering loss to follow-up and transfer-out as competing events [16].

Then, we analysed cause-specific mortality via competing risk analysis, where for each cause of death, loss to follow-up, transfer, and the other causes of death were considered as competing events. We used different approaches:

- We plotted cumulative incidence functions to analyse trends since start of follow-up
- We used univariable and multivariable competing risk regression to assess risk factors of different causes of death, with Fin and Gray regression models for each cause of death.

The following explanatory variables were included in the models: age category (<10, 10–19, and >19 years), sex, baseline CD4 category (<100, 100–349, 350–500 and >500 cells/mm3) baseline (at ART initiation), WHO stage of the disease (stage 1,2,3 or 4) and year of ART commencement (2004–2007, 2008–2011 and 2012–2017).

Baseline WHO stage at ART initiation was missing for a significant proportion of patients (34.7%). We used multiple imputation by chained equation (R package mice) to complete the missing data. Results from multiple imputation were combined using Rubin’s rule. We added the outcome, year of entry and presence of TB to improve the results of the imputation.

Competing risk analyses were done using the R packages survival and mstate. All other statistical analyses were done using Stata 13.1. All statistical tests were two-sided and variables with a p-value of 0.05 were considered statistically significant.

## Results

### Baseline characteristics

Between 1 February 2004 and 31 December 2017, 4 868 (62.1% female, n = 3 023) ART naïve patients were commenced on ART at NC and followed up for a median of 5.6 years (IQR: 2.2–8.5) with a total follow up time of 27 527 person-years (PY). Median age at ART commencement was 33 years (IQR 16–41). The median baseline CD4 cell count was 190 cells/mm^3^ (IQR:89–314). Table 2 summarises the baseline characteristics.

**Table 2:** Baseline characteristics at antiretroviral therapy initiation (N = 4868).

Characteristic	Frequency n (%)		
	Died	Alive	Total
Sex			
Female	300 (59.3)	2723 (62.4)	3023 (62.1)
Male	206 (40.7)	1639 (37.6)	1845 (37.9)
Age, median (IQR)	35 (17–47)	33 (16–40)	33 (16–41)
Age Groups			
Paediatrics (0–12 years)	79 (15.6)	864 (19.8)	943 (19.4)
Adolescents (13–19 years)	68 (13.4)	366 (8.4)	434 (8.9)
Adults (>19 years)	359 (70.9)	3132 (71.85)	3491 (71.7)
CD4 categories (cells/mm^3^)			
> 500	20 (4.0)	438 (10.0)	458 (9.4)
350–500	31 (6.1)	451 (10.3)	482 (9.9)
100–349	179 (35.4)	2365 (54.2)	2544 (52.3)
< 100	272 (53.8)	1069 (24.5)	1341 (27.5)
Clinical WHO Stage*			
1	27 (5.3)	778 (17.8)	805 (16.5)
2	50 (9.9)	737 (16.9)	787 (16.2)
3	173 (34.2)	988 (22.7)	1161 (23.8)
4	102 (20.2)	326 (7.5)	428 (8.8)
Pre-existing malignancy	16 (3.2)	27 (0.6)	43 (0.9)
Year of ART initiation			
2004–2007	238 (47.0)	1115 (25.6)	1353 (27.8)
2008–2011	175 (34.6)	1666 (38.2)	1841 (37.8)
2012–2017	93 (18.4)	1581 (36.2)	1674 (34.4)

*1687 Patients did not have a recorded WHO stage

### All-cause mortality

**Fig 1:**
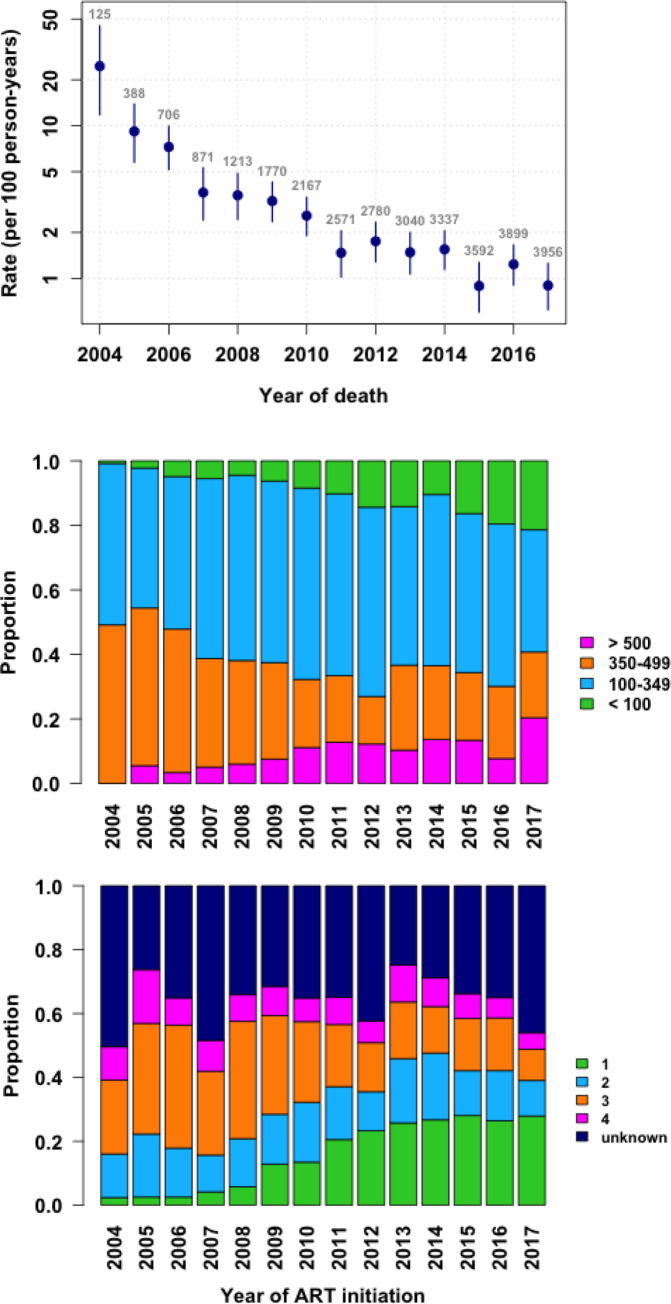
Trends in mortality rates per 100 person-years (with 95% confidence intervals; top panel), baseline CD4 count (middle panel) and WHO stage (lower panel) at Newlands Clinic 2004–2017: Cumulative Incidence Curve.

A total of 504 patients died from various causes during the period under review. Of these, 250 (49.4%) died within the first year of commencing ART. The median age at the time of death was 36 years (IQR: 19–46). The majority of patients were severely immunosuppressed at the time of death with a median CD4 cell count of 160 cells/mm^3^ (IQR: 41–357). The COD was TB which was responsible for 14.1% of the deaths. Meningitis accounted for 9.07% of deaths and 11.9% of deaths were due to NCDs (diabetes mellitus, renal failure and cardiovascular conditions). Different forms of malignancies caused 10.7% of the deaths. A specific cause of death could not be established for 15% of the patients. Table 3 highlights the distribution of the various causes of death. There was a significant decline in crude mortality rate over time as shown in Fig 1 top panel. Fig 1, middle and lower panels show the baseline WHO stage and of CD4 cell count of ART naive patients enrolled at NC during the period 2004 to 2017.

**Table 3:** Multivariable analysis for risk factors (Hazard Ratios & 95% Confidence Intervals) for death among patients enrolled at Newlands Clinic, 2004–2017.

Variable	4868 individuals included	All-causes (506 deaths)	Malignancies (55 deaths)	Tuberculosis (71 deaths)	Communicable AIDS related illness (except TB) (140 deaths)	Chronic non-communicable diseases (93 deaths)	Other (73 deaths)	Unknown (74 deaths)
Gender (0 missing)	Female Male	P = 1(ref) 1.04 (0.87–1.25)	P = 1(ref) 1.28 (0.74–2.19)	P = 1(ref) 1.33 (0.83–2.14)	P = 1(ref) 1.08 (0.77–1.52)	P = 1 1.9 (ref) 0.91(0.59–1.39)	P = 1(ref) 0.81 (0.5–1.33)	P = 1(ref) 1.02 (0.64–1.62)
Age (years) (0 missing)	>19 10–19 <10	P = 0.0061(ref) 1.53 (1.2–1.98)0.89 (0.7–1.2)	P = 0.27(ref) 1.5 (0.7–3.3)0.6 (0.2–1.5)	P = 0.6(ref) 0.9 (0.4–2.1)0.6 (0.3–1.3)	P = 0.15(ref) 1.5 (0.9–2.5)1.1 (0.7–1.8)	P = 0.055(ref) 0.99 (0.5–1.9)0.38 (0.2–0.8)	P = 0.39(ref) 1.3 (0.6–2.7)1.2 (0.6–2.3)	P = 0.0093(ref) 2.7 (1.5–4.9)1.4 (0.8–2.7)
Baseline CD4 (Cells/mm3) (43 missing)	>500 350–500 100–349 <100	P<0.001(ref) 1.5 (0.9–2.7)1.3 (0.8–2.1)2.7 (1.7–4.4)	P = 0.018(ref) 1.3 (0.2–8)1.3 (0.3–6)2.2 (0.4–10.5)	P<0.001(ref) 0.9 (0.1–6.5)1.3 (0.3–5.8)3.1 (0.7–14)	P<0.001(ref) 0.5 (0.2–1.5)0.7 (0.3–1.4)1.7 (0.8–3.6)	P<0.001(ref) 2.3 (0.6–8.3)1.2 (0.4–4.2)2.1 (0.6–7.2)	P<0.001(ref) 5.7 (0.7–49)4.2 (0.6–32)9.2 (1.2–17)	P = 0.015(ref) 3.5 (0.7–17)2.7 (0.6–12)3.6 (0.8–16)
Baseline WHO stage (1687 missing)	1 2 3 4	P<0.001(ref) 1.2 (0.8–1.8)2.4 (1.7–3.5)3.9 (2.7–5.7)	P = 0.018(ref) 0.7 (0.3–1.9)0.8 (0.3–1.9)2.3 (0.9–5.4)	P<0.001(ref) 1.4 (0.4–5.3)4.2 (1.4–12.4)7.1 (2.3–22)	P<0.001(ref) 1.6 (0.6–4.4)3.4 (1.4–8.3)5.6 (2.3–14)	P<0.001(ref) 1 (0.4–2.5)3.2 (1.5–6.8)3.4 (1.5–7.8)	P = 0.024(ref) 0.8 (0.3–1.9)1.1 (0.5–2.5)2.2 (1.0–5.2)	P = 0.0057(ref) 1.9 (0.6–5.6)3.4 (1.2–9.1)4 (1.4–11.9)
Year of ART Initiation (0 missing)	2004–2007 2008–2011 2012–2017	P<0.001(ref) 0.8 (0.7–1)0.7 (0.6–1)	P = 0.007(ref) 0.7 (0.4–1.2)0.7 (0.3–1.4)	P = 0.02(ref) 0.8 (0.5–1.3)0.7 (0.4–1.4)	P = 0.0013(ref) 0.9 (0.6–1.2)0.7 (0.4–1.1)	P<0.001(ref) 1.3 (0.8–2.1)1.1 (0.6–2.1)	P = 0.001(ref) 0.7(0.4–1.2)0.2 (0.1–0.5)	P<0.001(ref) 0.5 (0.3–0.9)1.2 (0.6–2.2)
Pre-existing malignancy (0 missing)	No Yes	P<0.001(ref) 2.4 (1.4–4.1)	X X x	X X x	X X x	X X x	X X x	X X X

In the multivariable model, the risk of death was similar for males and females (HR: 1.04, CI: 0.87–1.25)). When compared to adults, adolescents (HR 1.53, CI: 1.17–1.98) were at greater risk of death, and children (HR 0.89, CI: 0.68–1.17) were at similar risk of death. Low baseline CD4 cell count of less than 100 cells /mm^3^ and higher baseline WHO clinical stage (Stages 3 and 4) were shown to increase the risk of death. The year of enrolment had no significant impact on the risk of death. Table 3 highlights the independent risk factors associated with mortality.

### Cause-specific mortality

The 1-year cumulative incidence of death due to TB is of 0.9%, whereas it is of 1% for NCDs, and 0.5% for malignancies. After 12 years of follow-up, those cumulative incidences increased to 1.9% for TB, 2.9% for NCDs and 1.4% for malignancies

**Fig 2:**
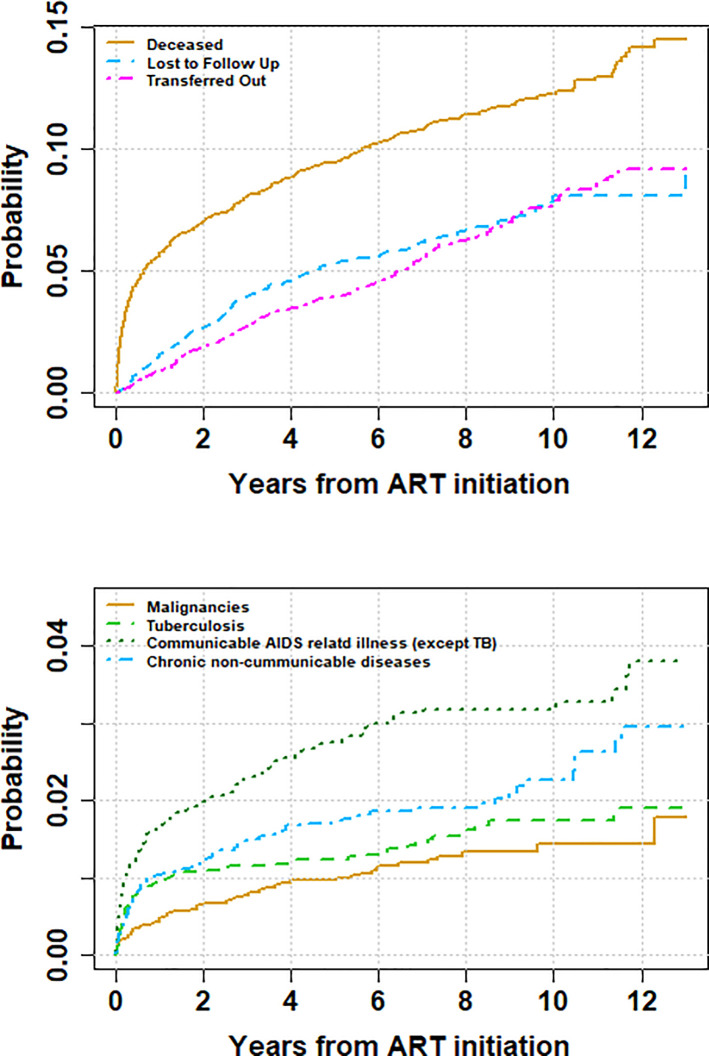
Cumulative Incidence of mortality, loss to follow up, transfer-out (upper panel) and cause specific mortality (lower panel).

Fig 2 upper panel shows cumulative incidence of death (all causes), LTFU and transfer and appendix 2 provides greater detail of the cumulative incidence of death in the first year of enrolment into care. Fig 2 lower panel highlights the cumulative incidence of the different causes of death For all the specific causes of death considered in our analysis, a low baseline CD4 cell count and higher baseline WHO clinical stage were shown to increase the risk of death. Neither age nor year of enrolment were shown to play a significant role in the risk of death, for all the specific causes of death investigated.

## Discussion

In this urban HIV clinic, our results show a steep decline in mortality rates over time from 2004 to 2017 which is in line with an improvement in the stage of disease and baseline CD4 cell counts. Communicable AIDS related illnesses, and in particular TB remained the leading cause of death over time. However, there was a significant increase in the probability of deaths related to malignancies and NCDs over time. Similar to other studies, we identified that most deaths were within a year of ART initiation into the NC HIV treatment program [17] and the major risk factors for death were poor baseline clinical and immunological status.

Our findings are consistent with the scientific literature showing that mortality in HIV positive people has declined in the past 20 years [1,18,19]. Several changes in the care and treatment of HIV-infected individuals have occurred over time and have impacted mortality rates. The Zimbabwe national HIV treatment program commenced in 2004. In 2004 access to ART was limited to a few sites nationally, leading to poor access. Over the years, access to ART has greatly improved to over 2000 sites. The periodic change in national treatment guidelines as advised by the WHO has led to earlier initiation of life saving ART [7]. Joint HIV and TB interventions and use of prophylactic treatment for common opportunistic infections i.e. cotrimoxazole prophylaxis and Isoniazid preventive therapy for TB have contributed to the sustained decrease in mortality rates [20]. Similarly lower mortality rates among HIV infected patients have been reported in other cohorts [3, 21]. The high mortality rates in our clinic are primarily because in the early years of the HIV epidemic, access to ART was poor, hence patients presented late for treatment.

Information on COD among patients on long term ART is scarce in SSA. Our results show that there is increasing mortality from malignancies and non-communicable diseases. The leading malignancies causing death were non-Hodgkin’s lymphoma, Kaposi sarcoma and cancer of the cervix. Among the NCDs, the leading causes of death were renal failure, cardiovascular diseases and diabetes mellitus.

With an increasing number of patients living longer with HIV, the incidence of chronic NCDs like hypertension and diabetes mellitus will rise [22]. This will require that HIV treatment programs reassess their traditional services. Screening for cervical cancer among women living with HIV must become part of routine care. Furthermore, it is essential that PLHIV have access to vaccination against Human Papilloma Virus, to prevent cervical cancer.

The median CD4 cell count closest to death was very low at 160 cells / mm^3^. We also identified low baseline CD4 cell count and WHO stage 3 and 4 as significant risk factors for death. This severe immunosuppression at death may explain why communicable AIDS related illnesses remained the major cause of death over time. This also highlights that many patients presented to our clinic late with advanced HIV infection and had a greater risk of death. Previous studies have shown that that such patients are at a high risk of dying of AIDS related communicable illnesses [9, 23]. Strategies to prevent late presentation with advanced immunosuppression need to be implemented. These should include timely detection of HIV infections, adequate linkage to HIV care and optimum monitoring of patients on treatment. Access to viral load monitoring will enable early detection of treatment failure and consequently timely referrals for specialist care.

TB caused death in 14% of patients and was the main cause of death across the study period. This is consistent with the findings of others, who have shown the major contribution of TB to death in the African HIV population [24–27].This finding highlights the need to strengthen HIV-TB collaborative services in settings such as Zimbabwe. There have been encouraging changes in the field of TB management in SSA which are likely to improve the clinical outcomes among HIV-TB co-infected people. Isoniazid preventive therapy, which has been shown to significantly reduce incidence of TB [28] has now been adopted by WHO as an essential package of care for HIV infected individuals. Furthermore, advances in TB diagnostics such as the Xpert MTB/RIF which has shown relatively high sensitivity in HIV infected patients are increasingly being implemented [29]. Early and better diagnosis of TB will lead to a reduction in TB associated morbidity and mortality among HV infected people. Our results showed that meningitis was the third leading cause of death in this cohort. The majority of cases of meningitis were due to *Cryptococcus neoformans* infection. The adoption of routine screening for cryptococcal infection among patients with a CD4 cell count below 100 cells/μl and pre-emptive therapy for cryptococcal meningitis will result in reduction of CM associated morbidity and mortality [30].

Our study had several limitations. COD based on clinic notes reviews are known to have relatively poor accuracy when compared to autopsy [10, 31]. This is a common problem for studies that use clinic notes reviews. We also acknowledge that assignment of causes of death in this study was not ideal nor was it very accurate. However, the assignment of COD reflects the real-world scenario in our setting, and we are convinced that our results represent the true changes in mortality patterns among HIV positive patients. The number of patients with unknown causes of death, 15%, was high partly because we could not link our clinic database to the national vital statistics register to obtain documented causes of death. Mortality may also have been underestimated due to LTFU and transfer out [13]. However, LTFU and transfer rates were relatively low throughout the follow up period. We do not expect that deaths among patients LTFU or transferred-out to significantly alter our results. We further acknowledge that our data could not allow us to assess the effects of ART initiation and duration of ART use on the risk of mortality. Despite these limitations, we believe that our results are representative of the mortality trends in our clinic and in Zimbabwe.

## Conclusion

Although the overall mortality rate among HIV infected patients declined over the past 13 years, this study found that the major causes of deaths were attributed to AIDS related illnesses. There remains a need for better screening strategies, accurate diagnostics and effective prevention strategies for TB and other common opportunistic infections. Furthermore, the contribution of NCDs and malignancies to mortality is significantly increasing. With a growing population on ART, one may assume that changes will continue in this direction. Innovative and accurate methods to assess death causes on a population level are needed, since complete autopsies are not widely available.

## Supporting information

S1 Appendix Leading causes of death at Newlands Clinic 2004–2017 (n = 506). (DOCX)

S2 Appendix Cumulative incidence death causes in year 1. (DOCX)

S1 Dataset (DTA)
