# Supplementary material for: Mortality trends and causes of death among HIV positive patients at Newlands Clinic in Harare, Zimbabwe
Source: PLoS One. 2020 Aug 27;15(8):e0237904. doi: 10.1371/journal.pone.0237904 (PMC7451579; doi:10.1371/journal.pone.0237904)
Supplement: S1 Appendix — (DOCX) [file pone.0237904.s001.docx]

**S1_Appendix. Leading causes of death at Newlands Clinic 2004-2017 (n=506).**

| **Cause of Death** | **Frequency** | **Percentage** |
| --- | --- | --- |
| Tuberculosis (TB) | 71 | 14 |
| Malignancy | 54 | 10.7 |
| Meningitis | 39 | 7.7 |
| Advanced HIV Infection | 39 | 7.7 |
| Diarrhoeal disease | 37 | 7.3 |
| Renal disease | 29 | 5.7 |
| Respiratory infection (excluding TB) | 24 | 4.7 |
| Cardiovascular Disease | 28 | 5.5 |
| Gastrointestinal and hepatic (excluding diarrhoea) | 13 | 2.6 |
| Diabetes Mellitus | 4 | 0.8 |
| Suicide | 5 | 1.0 |
| CNS conditions other than meningitis | 18 | 3.6 |
| Road traffic accident | 5 | 1.0 |
| Anaemia | 8 | 1.6 |
| Drug toxicity | 5 | 1.0 |
| Malaria | 3 | 0.6 |
| Psychiatric illness | 3 | 0.6 |
| Pregnancy related | 3 | 0.6 |
| Other illnesses | 42 | 8.3 |
| Unknown | 76 | 15 |
