# Supplementary figures and images for: Mortality trends and causes of death among HIV positive patients at Newlands Clinic in Harare, Zimbabwe
Source: PLoS One. 2020 Aug 27;15(8):e0237904. doi: 10.1371/journal.pone.0237904 (PMC7451579; doi:10.1371/journal.pone.0237904)

**Appendix 2: Cumulative incidence death causes in year 1**


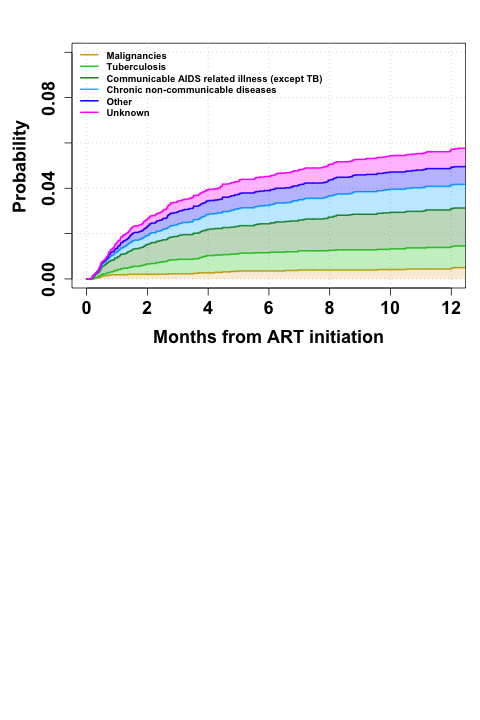

Supplement: S2 Appendix — (DOCX) [file pone.0237904.s002.docx]
